# Genome-Wide DNA Methylation Sequencing Identifies Methylation-Associated Genes Related to Porcine Skeletal Muscle Fiber Diversity

**DOI:** 10.3390/ijms27209093

**Published:** 2026-10-14

**Authors:** Chengwan Zha, Bojiang Li, Wen Yang, Liming Hou, Zhe Chao, Wangjun Wu

**Affiliations:** 1 Department of Animal Genetics, Breeding and Reproduction, College of Animal Science and Technology, Nanjing Agricultural University, Nanjing 210095, China; 2 College of Animal Science and Veterinary Medicine, Shenyang Agricultural University, Shenyang 110866, China; 3 Institute of Animal Science & Veterinary Medicine, Hainan Academy of Agricultural Sciences, Haikou 571100, China

**Keywords:** pig, muscle fiber, whole-genome bisulfite sequencing, DNA methylation, diseases

## Abstract

Alterations in skeletal muscle fibers are closely linked to human diseases, yet the mechanisms by which DNA methylation drives muscle fiber diversity remain unclear. Here, we performed whole-genome bisulfite sequencing (WGBS) to generate single-base-resolution DNA methylomes of fast-twitch *Biceps Femoris* (Bf) and slow-twitch *Soleus* (Sol) muscles and integrated transcriptomes to reveal associations between DNA methylation and gene expression. The global methylation level in skeletal muscle averaged 4.22%, with context-specific rates of 70.0% (CG), 1.16% (CHG), and 1.12% (CHH). The distribution of methylation levels of the mCs in CG and CHG contexts was similar but distinct from that in the CHH context. Furthermore, 152,286 DMCs and 51,079 DMRs were identified between Bf and Sol muscles. Bf showed higher global methylation (4.54%) than Sol (3.90%), and distinct differences in DNA methylation occurred throughout the genomic regions and gene functional element regions. CG-context DMCs and DMRs were mainly located in CpG islands and introns, while CHG and CHH DMCs and DMRs were exon-enriched. DMR-associated genes were significantly enriched in pathways related to muscle growth and remodeling and disease-related pathways. Moreover, five DMR-linked differentially expressed genes—*HSPB6*, *ATP2A2*, *TNNI1*, *HOXA11*, and *PTPN3*—were identified as critical candidate regulators of muscle fiber diversity. In addition, a context-dependent (CG, CHG, CHH) relationship between DNA methylation patterns and gene expression was observed across different genomic regions in the two muscle types. Our findings highlight the essential role of DNA methylation in skeletal muscle fiber diversity and identify potential epigenetic targets and pathways.

## 1. Introduction

Skeletal muscle is a type of striated muscle tissue that accounts for approximately 50% of body mass in mammals. Skeletal muscle is composed of various muscle fibers, and it exhibits a variety of physiological and metabolic properties due to the different proportions of specific muscle fiber types [1]. Fast-twitch fibers have strong glycolytic metabolic capacity and primarily express myosin heavy chain IIb (MyHC IIb), whereas slow-twitch fibers, which are enriched in mitochondria, have a higher oxidative capacity, and primarily express myosin heavy chain I (MyHC I) [1]. The types of adult muscle fibers and corresponding muscle properties can be altered by various external stimuli, and the conversion of muscle fiber types follows the rule of the “nearest neighbor”, namely, type I ↔ type IIa ↔ type IIx ↔ type IIb [2]. In humans, the diversity and plasticity of muscle fibers not only provide a wide range of functions to a given muscle mass but also lead to differential susceptibility to specific muscle diseases. It has been reported that many disorders and diseases, including muscle dystrophy, muscular atrophy, cardiomyopathic diseases, obesity, type 2 diabetes, aging, and other metabolic diseases, are profoundly affected by muscle fiber types [3,4,5]. Notably, we found many differentially expressed genes (DEGs) between the paired fast-twitch *Biceps Femoris* (Bf) and slow-twitch *Soleus* (Sol) from three pigs with the same genetic background using RNA-seq [6]. Therefore, we wondered whether Bf and Sol muscles from the same pigs had differentiated epigenomes that caused differences in gene expression, thereby leading to phenotypic differences in muscle fiber types.

DNA methylation is one of the major epigenetic modifications across species and is involved in the regulation of gene expression without altering the underlying DNA sequence. In mammals, DNA methylation occurs predominantly at the 5-position of cytosine within symmetrical CpG dinucleotides, although non-CpG methylation in CHG and CHH contexts (H = A, C, or T) also occurs [7,8]. DNA methylation, particularly in regulatory regions such as promoters, has been associated with transcriptional regulation in a context-dependent manner. Aberrant DNA methylation has also been implicated in various human diseases, including cancer, autoimmune diseases, metabolic disorders, aging, neurodegenerative diseases, and allergic diseases [9,10,11,12]. Epigenetic regulation has increasingly been recognized as an important molecular mechanism associated with skeletal muscle development and phenotypic variation in livestock. Previous studies in pigs and other farm animals have identified DNA methylation changes associated with skeletal muscle development, growth, muscle fiber characteristics, and meat-related traits, suggesting that DNA methylation represents an important regulatory layer underlying skeletal muscle phenotypic diversity in livestock [13,14,15,16]. Among the available approaches for DNA methylation profiling, bisulfite sequencing distinguishes methylated from unmethylated cytosines based on the bisulfite-mediated conversion of unmethylated cytosines to uracils, whereas methylated cytosines remain protected from conversion. Whole-genome bisulfite sequencing (WGBS) enables genome-wide profiling of DNA methylation at single-base resolution and is therefore well suited for comprehensively characterizing methylation differences between skeletal muscles with distinct phenotypic characteristics [17,18].

Relatively few studies have compared genome-wide DNA methylation profiles between porcine muscles with markedly different fiber-type compositions at single-base resolution. Here, we characterized DNA methylation profiles of Bf and Sol using WGBS and compared methylation patterns across CG, CHG, and CHH contexts. We further integrated the methylome data with previously generated transcriptomic data to identify critical candidate genes and pathways associated with muscle fiber types. Taken together, our data provide a basis for further investigation of DNA methylation associated with muscle fiber-type conversion and have potential implications for muscular diseases associated with altered muscle fiber types.

## 2. Results

### 2.1. The Difference of Immunofluorescence Staining Between Bf and Sol Muscles

Bf and Sol represent muscles enriched for fast- and slow-twitch fibers, respectively. Bf is characterized by higher glycolytic potential, whereas Sol is characterized by higher oxidative potential. A previous study has reported obvious differences in meat color between Bf and Sol muscles [6]. Consistently, in the present study, we observed significant differences in the types of muscle fibers between Bf and Sol, and the protein expression of *MYH7* (corresponding to type I fibers) was significantly greater in Sol than in Bf (Figure S1).

### 2.2. Overview of the WGBS Data

Six WGBS libraries (Bf28, Bf35, Bf36, Sol28, Sol35, and Sol36) were generated. Each library yielded more than 100 Gb of raw sequence, with Q30 values above 92.00%, and more than 84.0% of clean reads mapped to the reference genome. Mean genome-wide sequencing depth ranged from 20× to 28×, while mean cytosine coverage (C covgMean) ranged from 8× to 12×. The average bisulfite conversion rate of cytosines based on the lambda DNA genome reached 99.07%. Across all skeletal muscle samples, mean methylation of cytosines was approximately 4.22%. Context-specific averages were 70.0% for CG, 1.16% for CHG, and 1.12% for CHH. Bf consistently showed higher methylation than Sol across the three contexts: CG (70.57%, 70.72%, and 70.70% vs. 69.58%, 69.38%, and 68.82%), CHG (1.36%, 1.26%, and 1.84% vs. 1.13%, 0.68%, and 0.72%), and CHH (1.30%, 1.20%, and 1.77% vs. 1.10%, 0.65%, and 0.70%). The detailed data are shown in Table S1.

### 2.3. Genome-Wide DNA Methylation Characterization of Skeletal Muscles

**Figure 1:**
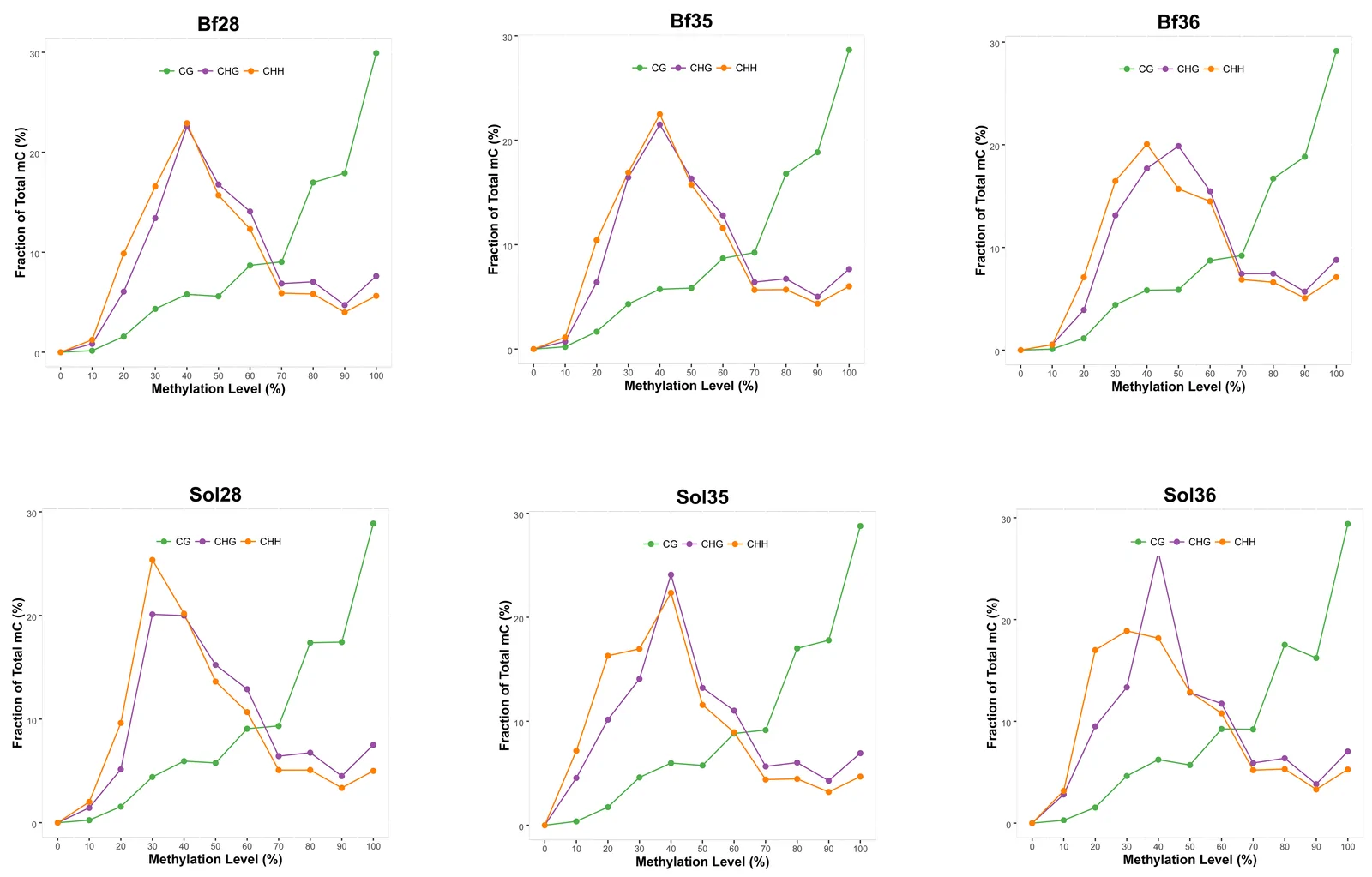
Distribution of methylation levels of the methylated sites under different contexts for each skeletal muscle. Different colors represent different contexts. The *X*-axis indicates the methylation level. The methylation level is divided into 10 segments with a 10% gradient. A methylation level of less than 5% is defined as 0%, a methylation level of greater than or equal to 95% is expressed as 100%, and the methylation in the middle is expressed as a middle value. The *Y*-axis represents the percentage of the methylated sites at a certain methylation level. The name of each sample is shown at the top of each plot. Bf: fast-twitch *Biceps Femoris*; Sol: slow-twitch *Soleus*.

Comparison of the paired samples showed that Bf contained more mCs than the corresponding Sol samples, with 37,667,145 and 37,366,389 mCs in Bf28 and Sol28, 41,661,421 and 38,487,554 in Bf35 and Sol35, and 40,711,047 and 32,788,008 in Bf36 and Sol36, respectively (Table S2). This pattern was consistent with the genome-wide methylation levels, which were higher in Bf (4.38%, 4.35%, and 4.88%) than in the corresponding Sol samples (4.21%, 3.75%, and 3.74%, respectively) (Table S1). In contrast, in the CHH context, more mCs were detected in Sol than in Bf (Table S2). Moreover, the majority of mCs were detected in the CG context, with the CHH and CHG contexts accounting for progressively smaller proportions. This pattern suggested that the genome-wide methylation differences between Bf and Sol might be primarily attributable to the greater number of CG-context mCs in Bf (Table S2). The distribution of methylation levels of the mCs in the CG and CHG contexts was similar but different from that in the CHH context (Figure 1).

The distribution of genome-wide methylation levels and densities among the skeletal muscle samples shows obvious differences between Bf and Sol samples in the CHG and CHH contexts (Figure S2). The genome-wide distribution of DNA methylation levels and densities across chromosomes for each skeletal muscle sample is presented as Circos plots in Figure S3. In the CHG (blue) and CHH (purple) contexts, chromosomal methylation levels displayed in the outer tracks showed greater variation in Sol than in Bf. These differences in methylation patterns suggest that altered DNA methylation may be associated with changes in gene expression, potentially contributing to the transition from fast- to slow-twitch muscle fibers.

The correlation analysis showed that the genome-wide methylation density and methylation level were highly significantly positively correlated in all contexts in Bf and Sol, especially in the CHG (*r* = 0.6795 in Bf with *p* < 0.0001; *r* = 0.7937 in Sol with *p* < 0.0001) and CHH (*r* = 0.7611 in Bf with *p* < 0.0001; *r* = 0.8906 in Sol with *p* < 0.0001) contexts (Figure S4). A significant positive correlation was also observed between the genome-wide gene density and methylation level in the CHG (*r* = 0.4798 in Bf with *p* < 0.0001; *r* = 0.2821 in Sol with *p* < 0.0001) and CHH (*r* = 0.4101 in Bf with *p* < 0.0001; *r* = 0.2078 in Sol with *p* < 0.0001) but not in the CG context, in Bf and Sol (Figure S5). A highly significant negative correlation existed between gene density and methylation density in the CG context in Bf (*r* = −0.6555 in Bf with *p* < 0.0001) and Sol (*r* = −0.6572 in Sol with *p* < 0.0001), but inconsistent results were observed in Bf and Sol in the CHG and CHH contexts; namely, a weak positive correlation between gene density and methylation density in the CHG (*r* = 0.1755, *p* < 0.0001) and CHH (*r* = 0.1535, *p* < 0.0001) contexts was observed in Bf, but no significant correlation was observed in Sol (Figure S6). Similarly, genome-wide transposable element (TE) density and methylation level showed highly significant negative correlations in all sequence contexts in Bf and Sol, especially in the CHG (*r* = −0.6013 in Bf with *p* < 0.0001; *r* = −0.5787 in Sol with *p* < 0.0001) and CHH (*r* = −0.5675 in Bf with *p* < 0.0001; *r* = −0.5048 in Sol with *p* < 0.0001) contexts (Figure S7). Notably, the correlation between TE density and methylation density displayed various patterns in different contexts (Figure S8). TE density and methylation density showed a significant positive correlation in the CG context (*r* = 0.1866 in Bf with *p* < 0.0001; *r* = 0.2201 in Sol with *p* < 0.0001), but a significantly negative correlation in CHG (*r* = −0.524 in Bf with *p* < 0.0001; *r* = −0.4846 in Sol with *p* < 0.0001) and CHH (*r* = −0.5219 in Bf with *p* < 0.0001; *r* = −0.4413 in Sol with *p* < 0.0001) contexts.

Furthermore, the distribution patterns of methylation in different genomic functional regions in Bf and Sol skeletal muscle were analyzed. In the CG context, methylation profiles across different genomic elements were highly similar between Bf and Sol, whereas such similarity was not observed in the CHG and CHH contexts (Figure S9a). Likewise, methylation profiles across the gene body and its 2 kb upstream and downstream regions were comparable between Bf and Sol in the CG context, but exhibited contrasting patterns between the two muscles in the CHG and CHH contexts (Figure S9b).

### 2.4. The Distinguished Genome-Wide Methylation Differences Between Fast- and Slow-Twitch Muscles

**Figure 2:**
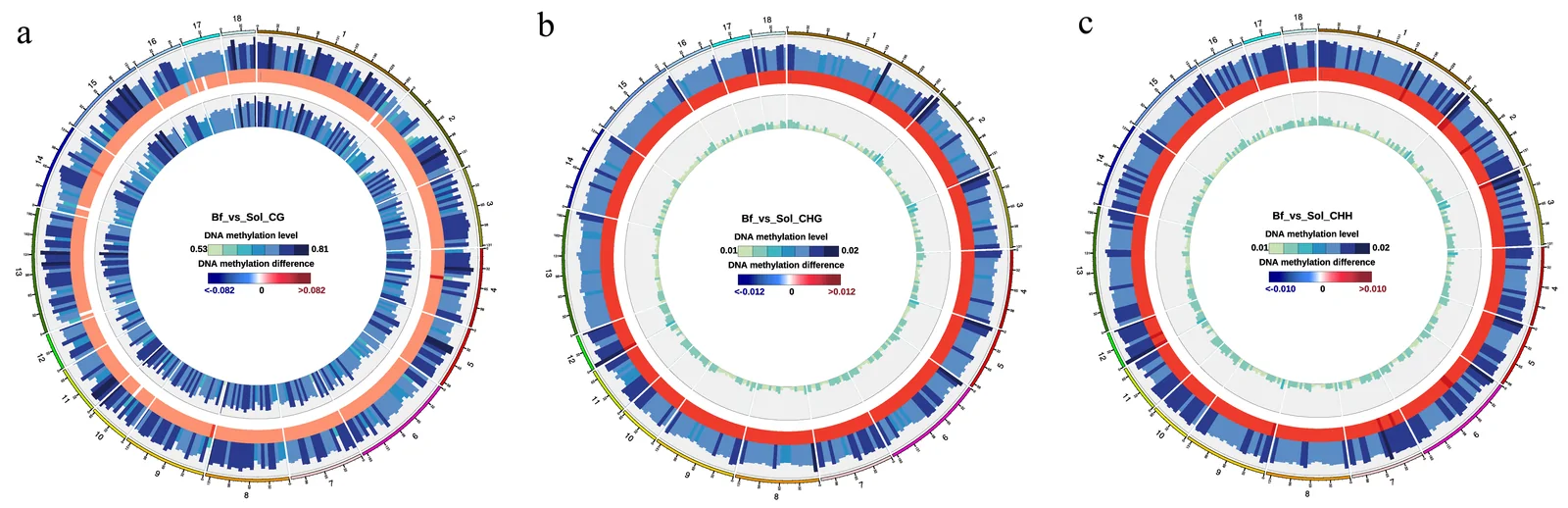
Circos plots of genome-wide methylation differences between Bf and Sol skeletal muscle. (**a**) Genome-wide methylation differences between Bf and Sol in the CG context; (**b**,**c**) genome-wide methylation differences between Bf and Sol in the CHG and CHH contexts, respectively. H is A, C, or T. The outermost circle represents chromosomes. The inner three circles, from outside to inside, represent the methylation level of the Bf group, the differences in the methylation levels between the sample groups, and the methylation level of the Sol group. Inner scale: the upper DNA methylation levels are indicated by color grading, and the lower heatmap indicates the degree of difference in methylation levels between samples. Bf: fast-twitch *Biceps Femoris*; Sol: slow-twitch *Soleus*.

We constructed a Circos plot to display the genome-wide methylation differences in three types of contexts between fast- and slow-twitch muscles (Figure 2). The genome-wide methylation profiles differed between Bf and Sol, with Bf showing higher overall methylation levels than Sol in the CG (Figure 2a), CHG (Figure 2b), and CHH (Figure 2c) contexts.

**Figure 3:**
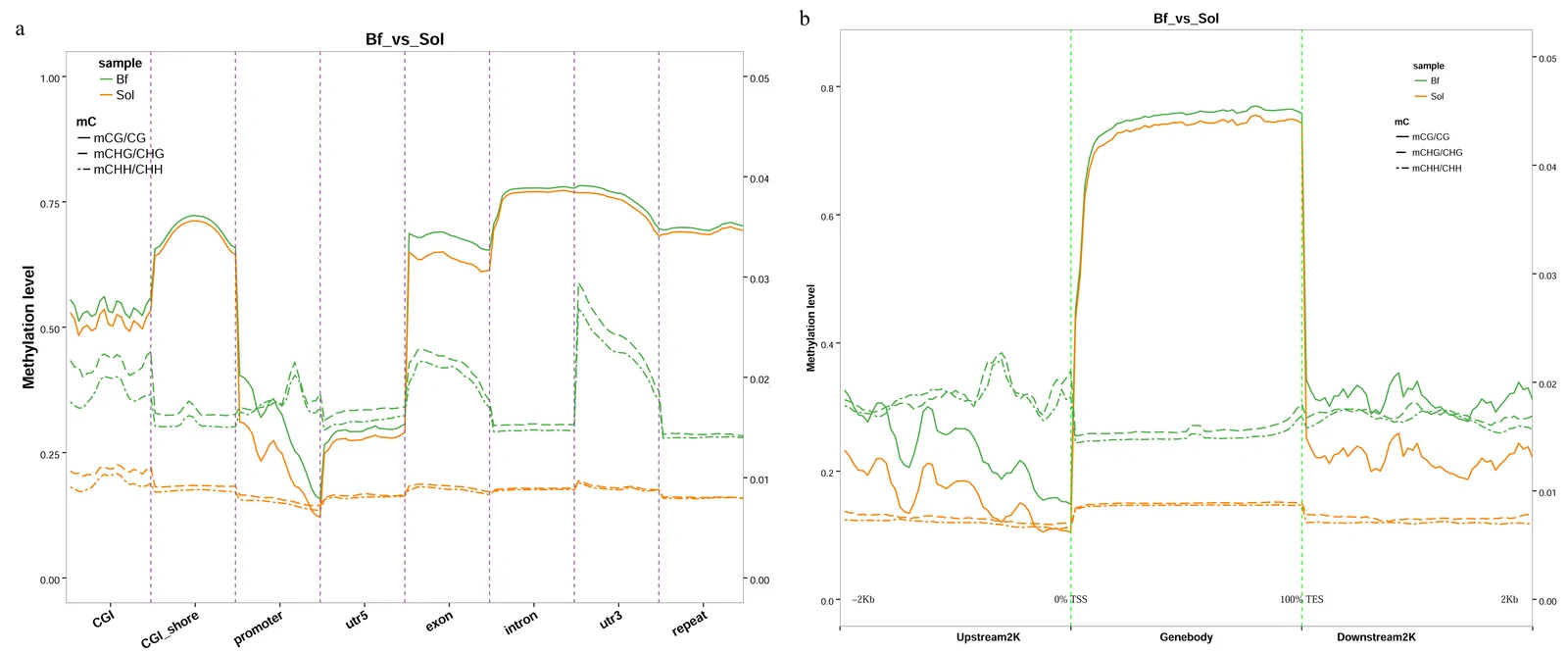
Methylation differences in different genomic regions between Bf and Sol skeletal muscle. (**a**) Comparative difference in methylation levels on the gene functional elements between the Bf and Sol groups under three types of sequence contexts. Different colors represent different groups, and different types of lines represent different sequence contexts (CG, CHG and CHH), where H is A, C, or T. The *X*-axis represents different gene genomic elements, and the *Y*-axis represents the methylation level. The left *Y*-axis displays the scale of methylation levels in the CG context; the right *Y*-axis displays the scale of methylation levels in the CHG and CHH contexts. Each functional region of each gene was equally divided into 20 bins, and then the average methylation level of C sites in the corresponding bin of all regions was calculated. CpG islands (CGIs) and CGIs shore were predicted based on the UCSC database (https://genome.ucsc.edu/) with a size greater than 200 bp, GC context greater than 50%, and expected value ratio > 0.6; CGI shore is a 2 kb region of CGI upstream and downstream; repeats were predicted using RepeatMasker (http://www.repeatmasker.org/); and the promoter region is a 2 kb region upstream of the transcription start site (TSS). (**b**) Comparative distribution of methylation levels in the regions 2 kb upstream and downstream of genes between the Bf and Sol groups. Different colors represent different groups, and different types of lines represent different sequence contexts (CG, CHG and CHH), where H is A, C, or T. The *X*-axis represents the different regions, and the *Y*-axis represents the methylation level. The left *Y*-axis displays the scale of methylation levels in the CG context; The right *Y*-axis displays the scale of methylation levels in the CHG and CHH contexts. Each region of each gene was equally divided into 50 bins, and then the average methylation levels of C sites in the corresponding bins of all regions were calculated. Different colors represent groups. TES: transcription termination site. Bf: fast-twitch *Biceps Femoris*; Sol: slow-twitch *Soleus*.

Across the three sequence contexts, methylation levels in different genomic regions were generally higher in Bf (light green) than in Sol (orange) (Figure 3). In the CG context, methylation levels exceeded 0.50 in most gene-associated functional regions, except for the promoter and 5′ UTR (Figure 3a). By contrast, in the CHG and CHH contexts, values for most functional regions were above 0.015 in Bf but below 0.010 in Sol (Figure 3a). Analysis of gene bodies and their flanking regions further showed that CG methylation was above 0.40 within gene bodies but below 0.40 in the 2-kb upstream and downstream regions (Figure 3b). For CHG and CHH methylation, levels across the gene body and both 2-kb flanking regions were above 0.015 in Bf and below 0.010 in Sol (Figure 3b).

### 2.5. Differentially Methylated Cytosines (DMCs) and Differentially Methylated Regions (DMRs) Associated with Skeletal Muscle Fiber Diversity

**Figure 4:**
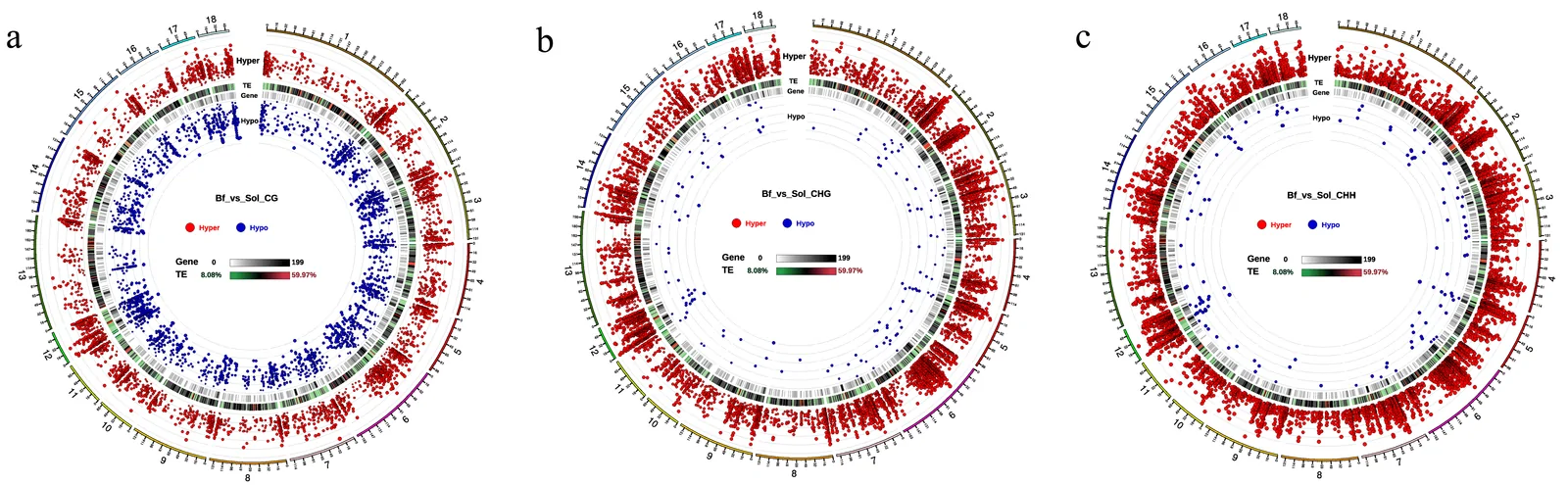
Genome distribution and significant differences in DMRs between Bf and Sol skeletal muscle. (**a**) Genomic distribution and significant differences in DMRs in the CG context; (**b**,**c**) genomic distribution and significant differences in DMRs in the CHG and CHH contexts, respectively. H is A, C, or T. Outer circles (from outside to inside): the outermost circle represents chromosomes; the second circle represents the genomic distribution and significant differences in the hypermethylated (Hyper) DMRs; the Hyper DMRs are indicated by red circles, and the Hyper DMR statistical value is expressed as log5 (|areaStat|); the higher and larger the outer dots, the more significant the differences; the third and fourth circles represent the transposable element (TE) ratio and the gene density heatmap, respectively (for the color indications, see the inner scales); the fifth circle represents the genomic distribution and significant differences in the hypomethylated (Hypo) DMRs, and the Hypo DMRs are indicated by blue circles, and the Hypo DMR statistical value is set as log5 (|areaStat|), the higher and the larger of the inward dots represent the more significant difference. Inner: the red dot represents the Hyper DMR and the blue dot represents the Hypo DMR; Gene represents the scale of gene number density, and the color gradient from gray to black indicates that the number of genes ranges from low to high; TE represents the heatmap of the TE ratio, and the color gradient from green through black to red indicates the ratio ranging from low to high. Bf: fast-twitch *Biceps Femoris*; Sol: slow-twitch *Soleus*.

The distinct genome-wide methylation patterns between fast- and slow-twitch muscles prompted us to identify DMCs. We identified a total of 152,286 DMCs between Bf and Sol, including 17,165 in the CG context (Figure S10a), 27,587 in the CHG context (Figure S10b), and 107,534 in the CHH context (Figure S10c). We further characterized the distribution of DMCs across different gene regions. In the CG context, DMCs occurred predominantly in CGIs, exons, and introns (Figure S11a). By comparison, CHG- and CHH-context DMCs exhibited distribution patterns distinct from those observed in the CG context, with both occurring primarily in exons and the 3′ UTR (Figure S11b,c). A total of 67,820 DMRs were annotated between Bf and Sol, comprising 13,056 CG, 25,379 CHG, and 29,385 CHH DMRs. Of the 13,056 DMRs identified in the CG context, 4975 were hypermethylated (Hyper) and 8081 were hypomethylated (Hypo). For the DMRs in the CHG and CHH contexts, most were Hyper DMRs (Figure 4). Subsequently, all the DMRs in the CG context and the most significant 10,000 DMRs in the CHG and CHH contexts were used for subsequent analysis. Detailed information on DMRs between Bf and Sol used for analysis is shown in Table S3, and the genomic distribution and significant differences in DMRs between fast- and slow-twitch muscles across various contexts were visualized using Circos plots (Figure 4).

**Figure 5:**
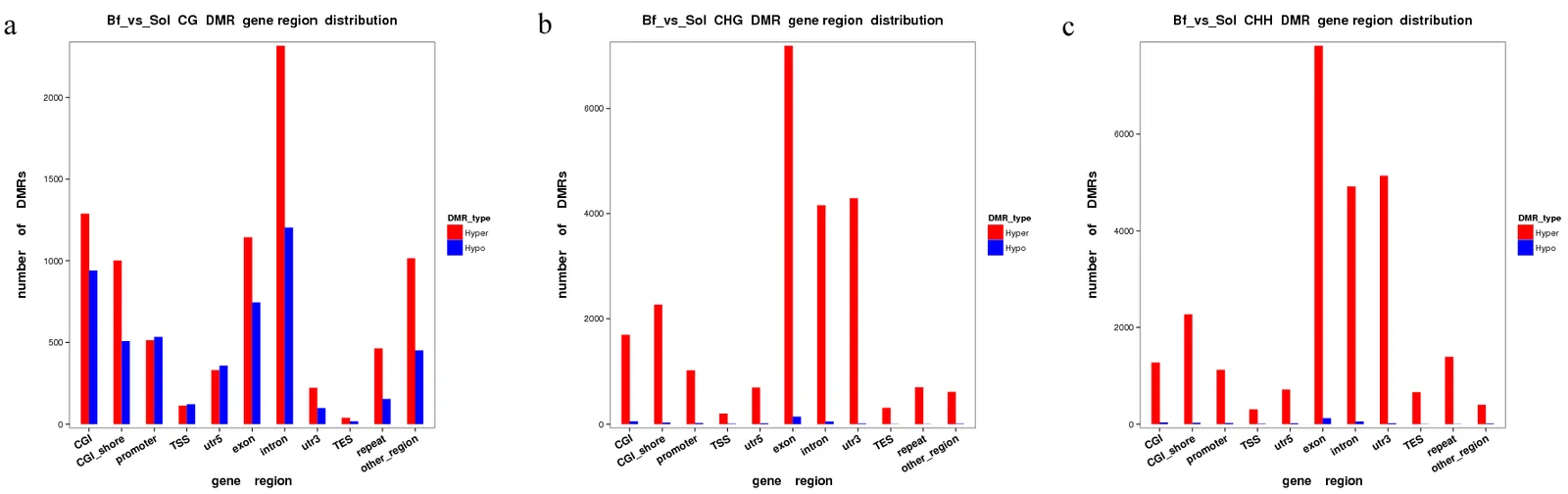
Comparative distribution of DMR-anchored regions between Bf and Sol skeletal muscle. (**a**) Distribution of DMR-anchored regions in the CG context; (**b**,**c**) Distribution of DMR-anchored regions in the CHG and CHH contexts, respectively. H is A, C, or T. The *X*-axis represents various functional regions, and the *Y*-axis represents the number of DMRs in each region. The functional regions are divided into promoters, exons, introns, CpG islands (CGIs), CGI shores (2 kb regions upstream and downstream of CGIs), repeat elements (repeat), transcription start sites (TSSs), and transcription termination sites (TESs). Red represents hypermethylated regions (Hyper), and blue represents hypomethylated regions (Hypo). Bf: fast-twitch *Biceps Femoris*; Sol: slow-twitch *Soleus*.

**Figure 6:**
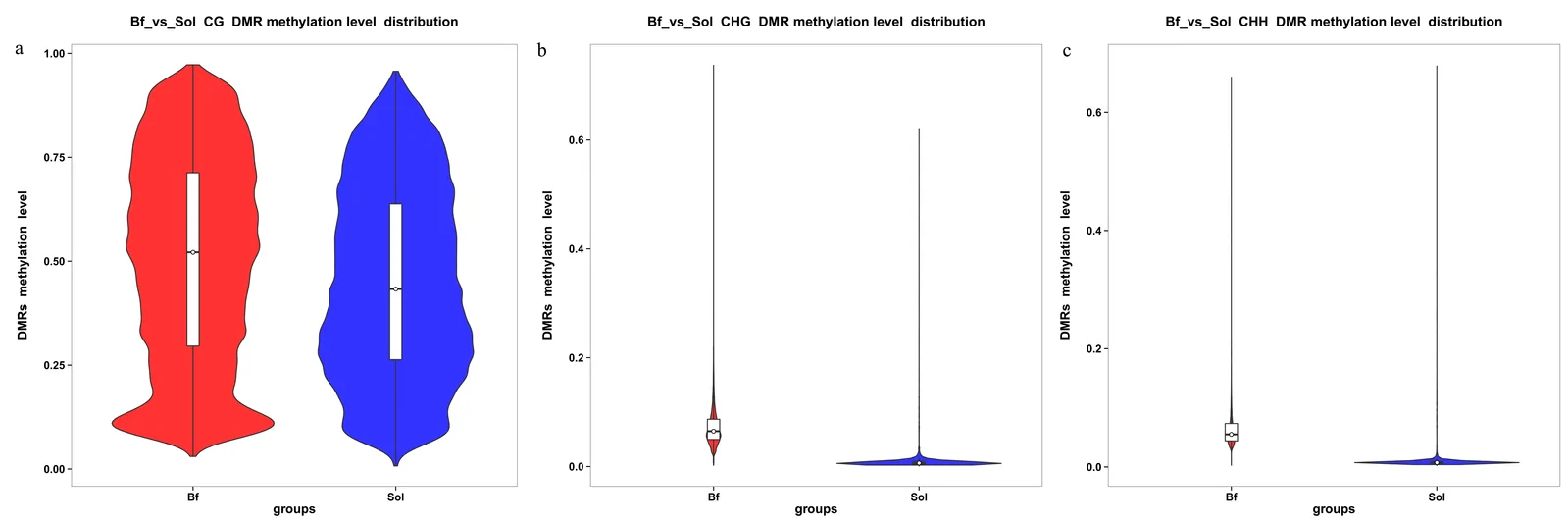
Comparative distribution of DMR methylation levels between Bf and Sol skeletal muscle. (**a**) Comparative distribution of DMR methylation levels in the CG context. (**b**) Comparative distribution of DMR methylation levels in the CHG context. (**c**) Comparative distribution of DMR methylation levels in the CHH context. CG, CHG, and CHH represent different sequence contexts, where H is A, C, or T. The *X*-axis represents the different groups, and the *Y*-axis represents the methylation level. The distribution of the DMR methylation level is displayed in the form of a violin plot, the inner plot is a boxplot, and the flanks are the number distribution under the methylation level. Bf: fast-twitch *Biceps Femoris*; Sol: slow-twitch *Soleus*.

We next characterized the length distribution of DMRs between the fast- and slow-twitch muscles. Most CG DMRs ranged from 50 to 200 bp, whereas CHG and CHH DMRs were predominantly distributed within the ranges of 50–500 bp and 100–1000 bp, respectively (Figure S12). We further examined the genomic distribution of DMRs across different gene regions and observed distinct patterns among the three sequence contexts (Figure 5). CG DMRs occurred primarily in intronic regions, whereas CHG and CHH DMRs were predominantly located in exons. In addition, Hyper DMRs constituted the predominant category across all three sequence contexts. Moreover, we examined the distribution of DMR methylation levels between the fast- and slow-twitch muscles. The median methylation level of DMRs was higher in Bf than in Sol (Figure 6), consistent with the greater number of DMRs showing higher methylation in Bf (Table S3).

**Figure 7:**
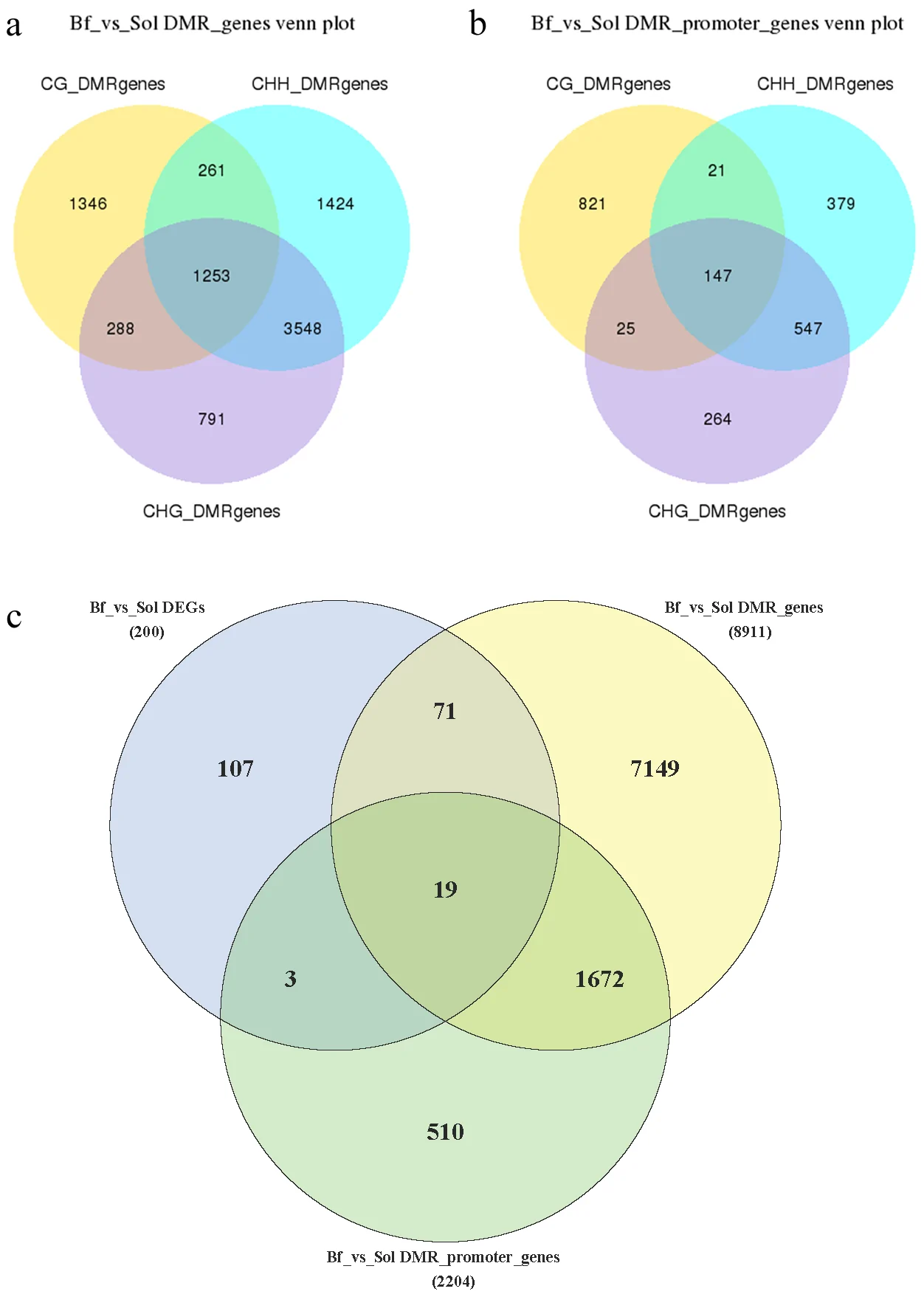
Venn diagram of DMR-anchored genes from Bf and Sol skeletal muscles. (**a**) Venn plot of DMR-anchored gene-body genes in different sequence contexts. An overlap greater than 1 bp between a DMR and a gene region (from TSS to TES) represents the anchor region, and the genes in this region are defined as the DMR-anchored gene-body genes. (**b**) Venn plot of DMR-anchored promoter region-related genes in different sequence contexts. The region where the overlap between the DMR and the 2 kb region upstream of the gene TSS is greater than 1 bp is the anchor region, and the genes in this region are defined as DMR-anchored promoter region-related genes. CG, CHG, and CHH represent different sequence contexts, where H is A, C, or T. (**c**) Venn plot of differentially expressed genes (DEGs), DMR-anchored gene-body genes, and DMR-anchored promoter region-related genes. TSS: transcription start site; TES: transcription termination site.

Additionally, we further characterized genes associated with DMRs in either gene bodies or promoter regions across the three sequence contexts. For gene-body DMRs, 15,514 gene associations were identified, comprising 3148 in the CG, 5880 in the CHG, and 6486 in the CHH context. After removing duplicates across contexts, these represented 8911 unique genes (Table S4a), of which 1253 were shared among all three contexts (Table S4b and Figure 7a). For promoter DMRs, 3091 gene associations were detected, including 1014, 983, and 1094 in the CG, CHG, and CHH contexts, respectively, corresponding to 2204 unique genes (Table S4c). Among these, 147 genes were common to all three contexts (Table S4d and Figure 7b).

To investigate the potential biological function of DMR-associated genes, GO and KEGG pathway enrichment analyses were conducted separately for genes with DMRs overlapping their gene bodies or promoters. In the CG context, the most significantly enriched GO terms were associated with biological processes, whereas cellular component terms showed the most significant enrichment in the CHG and CHH contexts (Tables S5 and S6). A KEGG analysis further revealed that ‘Pathways in cancer’ was the most significantly enriched pathway for both gene-body- and promoter-associated DMR genes in the CG context. In the CHG and CHH contexts, ‘Metabolic pathways’ was the most significantly enriched pathway for genes whose gene-body regions were anchored by DMRs, whereas ‘Ribosome’ was the most significantly enriched pathway for the genes whose promoter regions were anchored by DMRs. Notably, the significantly enriched ‘Pathways in cancer’ pathway contained the largest number of genes associated with DMR-anchored gene-body regions in the CG context, and the significantly enriched ‘Metabolic pathways’ contained the largest number of genes associated with DMR-anchored gene-body regions in both the CHG and CHH contexts. The significantly enriched ‘Metabolic pathways’ pathway contained the largest number of genes associated with the DMR-anchored promoter regions in the CG, CHG and CHH contexts. In addition, many important signaling pathways involved in skeletal muscle growth and remodeling were significantly enriched, such as ‘Calcium signaling pathway’, ‘Ras signaling pathway’, ‘MAPK signaling pathway’, ‘Hippo signaling pathway’, ‘Wnt signaling pathway’, ‘PI3K-Akt signaling pathway’, and ‘mTOR signaling pathway’. Moreover, many disease-related pathways, such as ‘Dilated cardiomyopathy’, ‘Alzheimer’s disease’, and ‘Parkinson’s disease’ were also significantly enriched. Detailed information on the significantly enriched pathways is shown in Tables S7 and S8, and Figure S13. Table S7 and Figure S13a–c show the pathways related to the genes whose gene bodies were anchored by DMRs, and Table S8 and Figure S13d–f show the pathways related to the genes whose promoters were anchored by DMRs.

### 2.6. Integrated Analysis of the DNA Methylome and Transcriptome

We further integrated the DNA methylome and transcriptome datasets to investigate the relationship between genomic DNA methylation and gene expression. Firstly, we performed intersection analysis using the DMR-anchored genes and DEGs (Table S9a) (FDR < 0.01) derived from our previous study [6]. Among these genes, 90 DEGs were associated with differential methylation of the gene body, 21 DEGs were associated with differential methylation of promoter, and 19 DEGs were simultaneously associated with methylation differences in the gene body and promoter (Table S9b,c and Figure 7c). Among these genes, the methylation levels of DMRs located in promoters of *HOXA11* and *TNNI1* were significantly higher in Bf skeletal muscles in CG context, corresponding lower gene expression levels. Likewise, the methylation levels of DMRs located in promoters of *ATP2A2*, *HSPB6* and *PTPN3* were significantly higher in Bf skeletal muscles in CHG context, corresponding lower gene expression levels. These results suggest that promoter hypermethylation was observed to be associated with lower expression among the candidate genes identified in this study. The visualization of the differential methylation status and expression of these five critical candidate genes is shown in Figure S14.

**Figure 8:**
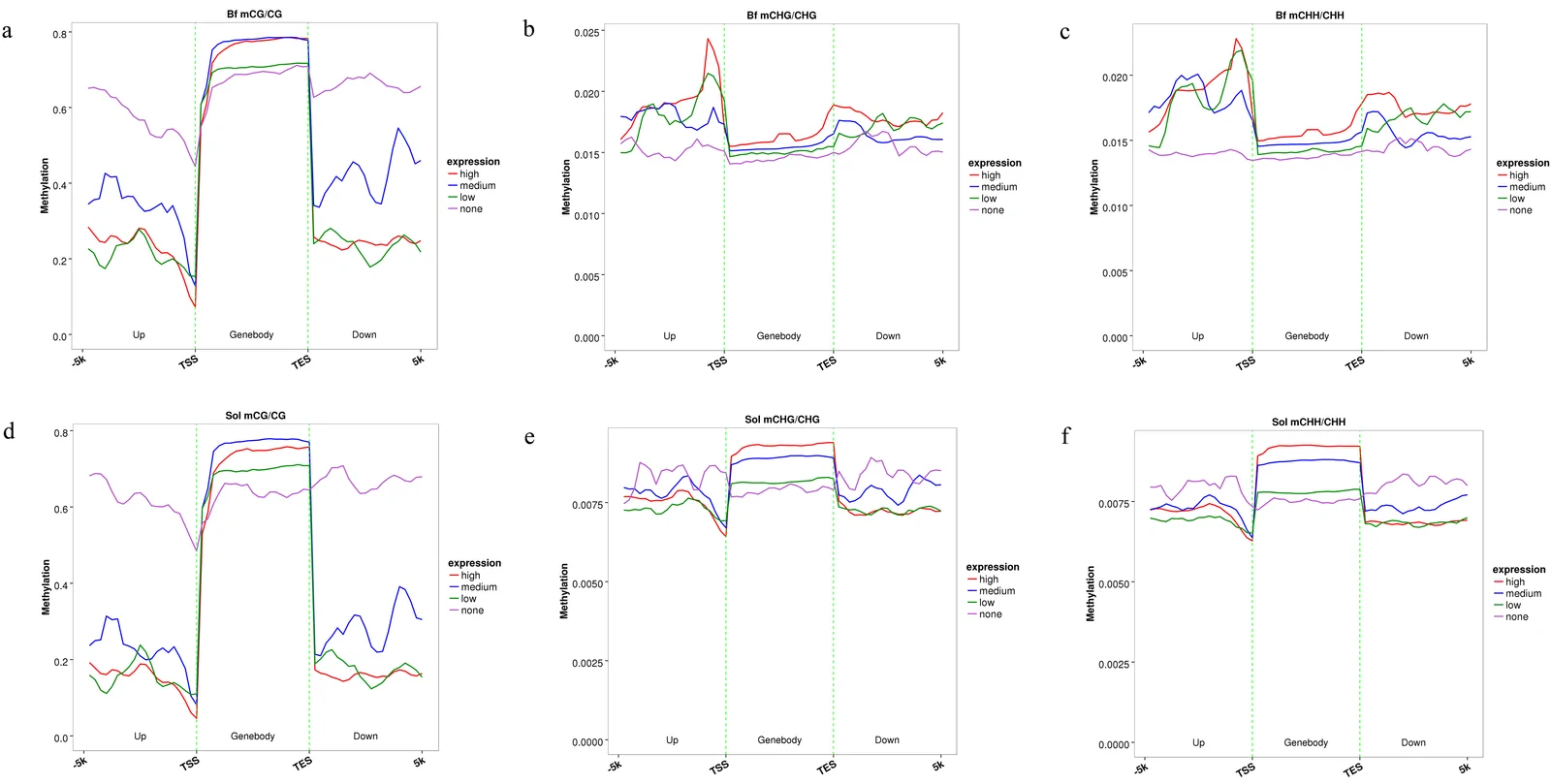
Relationship between gene expression and genomic methylation level. (**a**,**d**) Methylation patterns of four grouped genes in Bf and Sol in the CG context, respectively; (**b**,**e**) Methylation patterns of four grouped genes in Bf and Sol in the CHG context, respectively; (**c**,**f**) Methylation patterns of four grouped genes in Bf and Sol in the CHH context, respectively. H is A, C, or T. Red, blue, green, and purple lines represent highly expressed, intermediately expressed, lowly expressed, and unexpressed gene groups, respectively. The *X*-axis represents different gene genomic elements, and the *Y*-axis represents the methylation level. Each functional region of each gene was equally divided into 20 bins, and then the average methylation level of the C sites in the corresponding bin of all regions was calculated. Bf: fast-twitch *Biceps Femoris*; Sol: slow-twitch *Soleus*.

Based on transcriptomic expression levels, genes were classified into four groups: nonexpressed (none), high-, medium-, and low-expression genes, and their methylation profiles were subsequently examined in the CG, CHG, and CHH contexts. In the CG context, the four expression groups exhibited similar methylation profiles between Bf and Sol (Figure 8a,d). Methylation levels in the regions upstream and downstream of the gene body were higher in nonexpressed genes than in expressed genes, whereas the opposite pattern was observed within the gene body. Moreover, medium-expression genes showed higher CG methylation levels across all examined genomic regions than either high- or low-expression genes. In the CHG and CHH contexts, however, the methylation profiles of the four expression groups differed between Bf and Sol (Figure 8b,c,e,f). In Bf, nonexpressed genes exhibited lower methylation levels than the other expression groups across all examined genomic regions. By contrast, the methylation profile of nonexpressed genes in Sol resembled that observed for nonexpressed genes in the CG context in both Sol and Bf.

## 3. Discussion

Given the importance of muscle fiber types in human diseases and livestock meat quality, we explored the epigenetic regulatory mechanism of DNA methylation in muscle fiber diversity using the WGBS method based on a pig model. The Bf is a typical fast-twitch muscle, whereas the Sol is a typical slow-twitch muscle [6]. The paired design, in which Bf and Sol were measured in the same animals, minimized between-animal variation. Nevertheless, statistical power and the evaluation of inter-individual variability were constrained by the inclusion of only three full-sib female pigs. Future validation in larger independent cohorts and complementary experimental models is therefore required. In this study, both the total number of mCs and their overall methylation levels were higher in Bf than in Sol (Table S2 and Figure 2). This difference was accompanied by distinct methylation profiles across genomic features, including promoters, gene bodies, and regulatory elements (Figure 3), suggesting a potential role for DNA methylation in skeletal muscle fiber diversity. Interestingly, we observed a similar distribution of methylation levels among the mCs in the CG and CHG contexts, but inconsistent distribution patterns were found in the CHH context when compared with those in the CG and CHG contexts (Figure 1), indicating that the roles of cytosine methylation in the CHH context may be distinct from those of cytosine methylation in the CG and CHG contexts. In addition, a differential relationship between methylation density and level was observed across different contexts. The genome-wide methylation level and gene density were higher in the CG context than in the CHG and CHH contexts (Figure S3), which is consistent with the fact that methylation mainly occurs in the CG context in the animal kingdom.

Strikingly, the genome-wide methylation level was highly positively correlated with methylation density in all three types of contexts (Figure S4), indicating that the density of mCs is a critical factor affecting the methylation level. In the CG context, increasing gene density was associated with decreasing methylation density (Figure S6), whereas no significant relationship was detected between gene density and methylation level (Figure S5). By contrast, gene density was significantly correlated with methylation levels in the CHG and CHH contexts (Figure S6), suggesting that the relationship between gene density and DNA methylation varies according to sequence context. TE density was negatively correlated with methylation levels across all three contexts (Figure S8). However, its relationship with methylation density in the CG context was opposite to that observed in the CHG and CHH contexts (Figure S9). Collectively, these context-specific relationships suggest that cytosine methylation in different sequence contexts may have distinct biological roles.

DNA methylation is a classical epigenetic regulatory mechanism involved in gene transcription, X-chromosome inactivation, genomic imprinting, the regulation of germline-specific genes, and transposon repression [19]. Therefore, DNA methylation is of paramount importance for various biological events, including mammalian development, aging, and various diseases [19]. Moreover, DNA methylation is an important determinant of tissue- and cell-specific gene expression [20]. In the present study, genome-wide and genomic feature-based analyses consistently showed higher methylation levels in Bf than in Sol across all three sequence contexts (Figure 2 and Figure 3). Differences in muscle fiber composition are particularly relevant to a variety of pathological and physiological conditions, including muscular dystrophy, muscle atrophy, cardiomyopathy, obesity, type 2 diabetes, aging, and other metabolic disorders [3]. These observations support further investigation of whether muscle-associated methylation patterns are relevant to disease-related skeletal muscle phenotypes. In livestock, meat quality is directly affected by differences in skeletal muscle fiber types [21,22], further suggesting that DNA methylation-mediated epigenetic regulation may also contribute to economically important traits.

To further investigate the potential epigenetic role of DNA methylation in skeletal muscle fiber diversity, we characterized the associated differentially methylated cytosines (DMCs) and differentially methylated regions (DMRs). Notably, DMCs are widespread in various regions of the genome, but their predominant locations differ between the CG and the CHG or CHH contexts (Figure S11). The DMCs are mainly located in the CGIs, exons, and introns of genes in the CG context, whereas DMCs in both the CHG and CHH contexts are mainly located in exons and 3′ UTRs. There is no doubt that the methylation of functional genomic elements plays an important role in biological events. A classic example is the variation in intron 3 of *IGF2* located at an evolutionarily conserved CpG island, which can lead to a change in methylation status, thereby controlling muscle growth in pigs [23]. Therefore, the DMCs identified between the fast- and slow-twitch muscle fibers provide a good foundation for identifying the critical sites affecting muscle fiber diversity.

Given the potential regulatory roles of DMRs in gene transcription, we identified DMRs between Bf and Sol across the CG, CHG, and CHH contexts based on methylated cytosines. Our results indicated that the length and location of DMRs in the genome between Bf and Sol in the CG context showed obvious differences when compared with those in the CHG or CHH contexts, suggesting that the functions of DMRs derived from the CG context may be different from the functions of DMRs derived from the CHG or CHH contexts. Accordingly, we also found that different pathways were enriched in different contexts in the KEGG analysis (Tables S7 and S8). It is worth noting that some significantly enriched pathways involved in skeletal muscle growth and skeletal muscle fiber transformation were found (Tables S7 and S8). The ‘Calcium signaling pathway’ and the ‘MAPK signaling pathway’ are known to be critical for skeletal muscle remodeling [24]. Consistently, we found that both pathways were enriched among genes associated with DMRs, including those located in gene bodies and promoters. In addition, the significantly enriched pathways ‘Ras signaling pathway’ [25], ‘Hippo signaling pathway’ [26], ‘Wnt signaling pathway’ [27], ‘PI3K-Akt signaling pathway’ [28], ‘mTOR signaling pathway’ [29], and ‘cAMP signaling pathway’ [30] were demonstrated to play important roles in skeletal growth and development. Taken together, these results indicate that the genes anchored by DMRs are critical candidates for skeletal growth and development and skeletal muscle fiber transformation. Notably, some disease-related pathways, including the ‘Pathways in cancer’, ‘Proteoglycans in cancer’, ‘HTLV-I infection’, ‘Dilated cardiomyopathy’, ‘Alzheimer’s disease’, ‘Parkinson’s disease’, ‘Insulin signaling pathway’, and ‘Longevity regulating pathway’ were significantly enriched. Among these, ‘Pathways in cancer’ showed the most significant enrichment in the KEGG analyses of genes associated with both gene-body and promoter DMRs in the CG context. These findings provide further insight into the potential involvement of DMR-associated genes in disease-related biological processes, particularly those relevant to cancer.

Based on the genome-wide gene expression profiles of porcine fast-twitch Bf and slow-twitch Sol muscle [6], we further explored the relationship between gene expression and methylation level by integrating the present genome-wide methylation data. In this study, we found 19 critical intersected genes between the DMR-anchored genes and DEGs. Among these genes, *HSPB6*, *ATP2A2*, *TNNI1*, *HOXA11*, and *PTPN3* showed significant differences in both promoter DMR methylation and expression, with higher methylation corresponding to lower gene expression (Figure S14). *HSPB6*, a 17-kDa member of the small heat shock protein family, is constitutively expressed at high levels in smooth, cardiac, and skeletal muscles and is involved in muscle function and muscle-related diseases [31,32]. *ATP2A2*, encoding sarcoendoplasmic reticulum Ca^2 +^-ATPase 2 (SERCA2), is a key gene involved in intracellular Ca^2+^ homeostasis and is related to slow muscle formation [33], and recent reports have indicated that it is associated with occurrence of muscle-related diseases [34,35]. *TNNI1*, encoding the slow skeletal muscle isoform of Troponin I (TnI), plays a central role in the calcium regulation of contraction and relaxation [36]. HOXA11 is a critical regulator in myogenic precursors involved in the negative regulation of MyoD and related to slow-twitch muscle formation [37,38]. A recent study indicated that PTPN3 is a novel biomarker for predicting cancer prognosis, immunity, and immunotherapeutic efficacy [39]. Together, these results support the identification of *HSPB6*, *ATP2A2*, *TNNI1*, *HOXA11*, and *PTPN3* as candidate genes involved in muscle fiber diversity, and the observed methylation differences point to a potential epigenetic mechanism influencing muscle metabolism.

Previously, many studies focused on the effect and regulatory mechanism of promoter methylation on gene expression, and it is well known that the methylation of gene promoters suppresses gene expression and contributes to the tissue- and cell-specific expression of genes [20]. Consistent with this regulatory pattern, our results revealed a negative relationship between methylation upstream of the gene body and gene expression in the CG context (Figure 8a,b). By contrast, gene-body methylation was positively associated with gene expression in the CG context (Figure 8a,b), which is consistent with previous reports [40]. Therefore, the underlying roles and regulatory mechanisms of DNA methylation at the gene body may be different from those at the promoter. Interestingly, in the CHG and CHH contexts, the negative relationship between methylation in the upstream or downstream regions and gene expression was observed in Sol but not in Bf. A positive association between gene-body methylation and expression was observed across all sequence contexts in the skeletal muscle samples. Furthermore, the four gene-expression groups exhibited distinct global methylation patterns between Bf and Sol in the CHG and CHH contexts. Collectively, these observations suggest that CHG and CHH methylation exhibit distinct muscle-associated patterns and relationships with gene expression between Bf and Sol, although their functional significance requires further investigation.

## 4. Materials and Methods

### 4.1. Animal and Skeletal Muscle Samples

Two types of skeletal muscles with different muscle fiber phenotypes, fast-twitch Bf and slow-twitch Sol, derived from three full-sib female Duroc × Meishan pigs were collected for the genome-wide methylation sequencing. Meanwhile, the Bf and Sol muscles, fixed in 4% paraformaldehyde, were used for immunofluorescence staining to assess the phenotypic differences of type I and type II fiber using the myosin heavy chain 7 (MYH7, DSHB, BA-D5) and MYH4 (DSHB, BF-F3) antibodies, respectively. All animal procedures were approved by the Institutional Animal Care and Use Committee of Nanjing Agricultural University (Approval No. NJAULLSC2022069). The detailed animal sources, sampling procedures, and phenotypic differences can be found in Li et al. [6].

### 4.2. Library Preparation and Genome-Wide DNA Methylation Sequencing

Genomic DNA was extracted from the two types of skeletal muscles mentioned above using the phenol: chloroform: isoamyl alcohol method. Before DNA extraction, the tissues were homogenized in liquid nitrogen. The integrity of genomic DNA was monitored on 1% agarose gels, the purity was checked using a Kaiao K5500^®^ Spectrophotometer (Kaiao, Beijing, China), and the concentration was measured using a Qubit^®^ DNA Assay Kit in a Qubit^®^ 2.0 Fluorometer (Life Technologies, Carlsbad, CA, USA). The qualified DNA was fragmented randomly by sonication to an average size of 200 bp with a Covaris S220 focused ultrasonicator (Covaris, Woburn, MA, USA) and then subjected to end repair, adenylation, and ligation of cytosine-methylated adapters. These DNA fragments were treated with bisulfite using an EZ DNA Methylation-Gold^TM^ Kit (Zymo Research, Irvine, CA, USA) according to the manufacturer’s protocols, and then the sequencing libraries were completed through PCR amplification of single-strand DNA fragments using KAPA HiFi Hotstart Uracil + ReadyMix (Kapa Biosystems, Wilmington, MA, USA). The concentration of sequencing libraries was first quantified by a Qubit^®^ DNA Assay Kit in a Qubit^®^ 2.0 Fluorometer (Life Technologies, Carlsbad, CA, USA), and the size of the insert fragments was checked using an Agilent Bioanalyzer 2100 system (Agilent, Technologies, Inc. Wilmington, DE, USA). Then, the accurate concentration of libraries was determined using the Q-PCR method on a StepOnePlus^TM^ Real-Time PCR system (Thermo Fisher Scientific, Carlsbad, CA, USA). Subsequently, the libraries were sequenced on an Illumina HiSeq platform with 150 bp paired-end reads. The WGBS sequencing data generated for this study have been deposited in the NCBI BioProject database with the accession code PRJNA902114.

### 4.3. Processing and Alignment of Sequencing Data

Raw data were generated by the Illumina pipeline in FastQ format and first preprocessed using Trimmomatic software (Trimmomatic v0.36), and high-quality clean reads were obtained by removing the adapter and the invalid reads containing poly-N and low-quality reads, and basic read statistics were conducted using the FastQC (fastqc_v0.11.5). Reference genome (*Sus scrofa* 11.1) mapping was performed using Bismark software (version 0.16.3) with the default parameters [41]. First, the normal reference genomic sequence was transformed into a bisulfite-converted version according to the principle of C to T and G to A and then indexed using Bowtie 2 [42]. Then, the sequenced reads were also transformed into fully bisulfite-converted versions according to the principle of C to T and G to A, and the unique best alignment reads were obtained through alignment analysis between converted sequenced reads and converted reference genomic sequences in a directional manner. Meanwhile, the methylation state of all cytosine positions in the read was inferred according to the alignment analysis between the unique best alignment reads and normal genomic sequence. The sequencing depth and coverage were calculated using deduplicated reads based on the unique mapping reads. The sodium bisulfite nonconversion rate was calculated as the percentage of cytosine sequenced at cytosine reference positions in the lambda genome.

### 4.4. Analysis of DNA Methylation Characteristics

The true methylation cytosines (mCs) were tested based on the binomial distribution method, and sites with read coverage ≥ 5, methylation level ≥ 0.05, and FDR ≤ 0.01 were defined as methylated sites. To calculate the methylation level of the sequence, we divided the sequence into multiple bins, with a bin size of 10 kb. The sum of methylated and unmethylated read counts in each window was calculated. The methylation level (ML) for each window or cytosine was calculated according to previous studies [43] as follows:

$$\mathrm{ML}\left(C\right)=\frac{\mathrm{reads}\left(\mathrm{mC}\right)}{\mathrm{reads}\left(\mathrm{mC}\right)+\mathrm{read}\left(C\right)}$$

Furthermore, the ML was corrected with the bisulfite nonconversion rate according to previous studies [44]. Given the bisulfite nonconversion rate *r*, the calculation formula of corrected ML was as follows:

$${\mathrm{ML}}_{\left(\mathrm{Corrected}\right)}=\frac{\mathrm{ML}-r}{1-r}$$

Moreover, the genome-wide distribution characteristics of mCs and single cytosine methylation levels in three different sequence contexts (CG, CHH, and CHG, where H represents A, C, or T) were analyzed. The differences in methylation level in each methylated site were calculated using DSS software [45], and the differential methylation sites were defined according to the following standards: difference in methylation level > 0.1 and the Posterior Probability of Differential Methylation (PPDE) > 0.95.

Furthermore, the genome-wide methylation level and density in three types of sequence contexts were analyzed, and the correlation was conducted between the methylation level and density. Methylation density was calculated separately for the CG, CHG, and CHH sequence contexts within each genomic bin. For each context, methylation density was defined as the number of methylated cytosine sites divided by the total number of cytosine sites of the corresponding context with sequencing coverage ≥ 5× within the same bin. A Circos plot of the distribution of the methylation levels and density on chromosomes was constructed according to a previous method [46]. Moreover, the methylation levels at different genomic elements, including promoters, exons, introns, CpG islands (CGIs), regions 2 kb upstream and downstream of CpG islands (CGI shore), repeat regions, and 5′ and 3′ untranslated regions (UTRs), and the methylation levels 2 kb upstream and downstream of the gene body were evaluated among the samples. In addition, visual Circos plots showing the genome-wide methylation level differences between samples were created, and a comparative analysis of the methylation level distribution at specific gene functional regions was conducted.

### 4.5. Analysis of Differentially Methylated Regions (DMRs)

The differentially methylated regions (DMRs) in three types of sequence contexts were detected using DSS software [45] according to three factors, namely, the spatial correlation between the neighboring mCs, the read depth of the cytosines and the variance among the biological replicates. The DMR identification parameters in DSS were as follows: smoothing = TRUE, smoothing.span = 200, delta = 0, p.threshold = 1 × 10^−5^, minlen = 50, minCG = 3, dis.merge = 100, and pct.sig = 0.5. When the number of DMRs in various sequence contexts (CG, CHG, and CHH) was more than 10,000, only the 10,000 DMRs with higher significance values were selected for subsequent analysis. The length distribution and methylation levels of DMRs between different groups were analyzed, and the gene functional regions anchored by DMRs were counted in the hypermethylated (Hyper) and hypomethylated (Hypo) regions, respectively. For the Bf versus Sol comparison, Hyper DMRs were defined as regions showing higher methylation levels in Bf than in Sol, whereas Hypo DMRs were defined as regions showing lower methylation levels in Bf than in Sol. Moreover, visual Circos plots of the distribution of DMRs across the genome were constructed. In addition, according to the distribution of DMRs throughout the genome, we defined the genes related to DMRs as genes whose gene-body region (from TSS to TES) or promoter region (upstream 2 kb from the TSS) overlapped with the DMRs.

### 4.6. Gene Ontology (GO) and KEGG Pathway Analysis

GO enrichment analysis was conducted with the DMR-anchored gene body and promoter genes, respectively, using the GOseq R package. The resulting *p* values were adjusted for multiple testing using the Benjamini–Hochberg (BH) false discovery rate (FDR) method. GO terms with FDR values less than 0.05 were considered significantly enriched. Pathway enrichment analysis was carried out using the KEGG database (http://www.genome.jp/kegg/, accessed on 5 September 2025), and KOBAS software Version 2.0 was used to test the statistical enrichment of DMR-related genes in KEGG pathways. Pathways with FDR values less than 0.05 were defined as significantly enriched.

### 4.7. Joint Analysis of the DNA Methylome and Transcriptome

Previously, genome-wide gene expression profiles of porcine fast-twitch-enriched Bf and slow-twitch-enriched Sol muscles were generated by RNA-seq, and differentially expressed genes (DEGs) were identified. Importantly, the RNA-seq and WGBS data were generated from Bf and Sol muscle samples collected from the same three experimental pigs [6]. To explore the relationship between gene expression and methylation levels, we conducted an overlapping genes analysis of the DMR-anchored gene-body genes and DMR-anchored promoter region-related genes in three different sequence contexts, and the DEGs. Furthermore, the methylation status of DMRs located in promoters and the expression of the critical intersected genes were visualized using IGV [47]. In addition, we divided the genes identified in transcriptome data into four groups according to gene expression levels (RPKM). The nonexpressed genes were set as a group, and others were equally divided into high, medium, and low groups. Furthermore, we analyzed the methylation patterns of these grouped genes between Bf and Sol muscle under three sequence contexts (CG, CHG, and CHH). The RNA-seq data used in this article are available in the NCBI BioProject database under the accession code PRJNA896756.

## 5. Conclusions

In summary, we established the genome-wide DNA methylation profiles of skeletal muscles with various muscle fiber types and revealed that the DNA methylomes are quite different between fast- and slow-twitch skeletal muscles. Furthermore, we identified the potential DMR-anchored genes and pathways involved in skeletal muscle fiber diversity. Additionally, we found that the DNA methylation status of different genomic functional regions was closely related to the gene expression and the regulatory differences depend on the CG, CHG, and CHH contexts. Given the importance of skeletal muscle diversity in many human diseases, our data could potentially contribute to therapies for human muscular diseases.

## Data Availability

The data presented in this study are openly available in NCBI BioProject database, reference number PRJNA902114 and PRJNA896756.

## Supplementary Materials

The following supporting information can be downloaded at: https://www.mdpi.com/article/10.3390/ijms27209093/s1.

## References

[1] Schiaffino S., Reggiani C. (2011). Fiber types in mammalian skeletal muscles. Physiol. Rev..

[2] Olsen L.A., Nicoll J.X., Fry A.C. (2019). The skeletal muscle fiber: A mechanically sensitive cell. Eur. J. Appl. Physiol..

[3] Talbot J., Maves L. (2016). Skeletal muscle fiber type: Using insights from muscle developmental biology to dissect targets for susceptibility and resistance to muscle disease. Wiley Interdiscip. Rev.-Dev. Biol..

[4] Ning H.Y., Ren H.W., Zhao Y., Yin H.F., Gan Z.J., Shen Y.J., Yu Y. (2023). Targeting the DP2 receptor alleviates muscle atrophy and diet-induced obesity in mice through oxidative myofiber transition. J. Cachexia Sarcopenia Muscle.

[5] Kim A., Koo J.H., Jin X., Kim W., Park S.Y., Park S., Rhee E.P., Choi C.S., Kim S.G. (2021). Ablation of USP21 in skeletal muscle promotes oxidative fibre phenotype, inhibiting obesity and type 2 diabetes. J. Cachexia Sarcopenia Muscle.

[6] Li B.J., Dong C., Li P.H., Ren Z.Q., Wang H., Yu F.X., Ning C.B., Liu K.Q., Wei W., Huang R.H. (2016). Identification of candidate genes associated with porcine meat color traits by genome-wide transcriptome analysis. Sci. Rep..

[7] Ramsahoye B.H., Biniszkiewicz D., Lyko F., Clark V., Bird A.P., Jaenisch R. (2000). Non-CpG methylation is prevalent in embryonic stem cells and may be mediated by DNA methyltransferase 3a. Proc. Natl. Acad. Sci. USA.

[8] Ziller M.J., Müller F., Liao J., Zhang Y.Y., Gu H.C., Bock C., Boyle P., Epstein C.B., Bernstein B.E., Lengauer T. (2011). Genomic Distribution and Inter-Sample Variation of Non-CpG Methylation across Human Cell Types. PLoS Genet..

[9] Legaki E., Taka S., Papadopoulos N.G. (2023). The complexity in DNA methylation analysis of allergic diseases. Curr. Opin. Allergy Clin. Immunol..

[10] Kaur G., Rathod S.S.S., Ghoneim M.M., Alshehri S., Ahmad J., Mishra A., Alhakamy N.A. (2022). DNA Methylation: A Promising Approach in Management of Alzheimer’s Disease and Other Neurodegenerative Disorders. Biology.

[11] Yazar V., Dawson V.L., Dawson T.M., Kang S.U. (2023). DNA Methylation Signature of Aging: Potential Impact on the Pathogenesis of Parkinson’s Disease. J. Park. Dis..

[12] Papanicolau-Sengos A., Aldape K. (2022). DNA Methylation Profiling: An Emerging Paradigm for Cancer Diagnosis. Annu. Rev. Pathol.-Mech. Dis..

[13] Yang Y.L., Fan X.H., Yan J.Y., Chen M.Y., Zhu M., Tang Y.J., Liu S.Y., Tang Z.L. (2021). A comprehensive epigenome atlas reveals DNA methylation regulating skeletal muscle development. Nucleic Acids Res..

[14] Fan Y.X., Liang Y.X., Deng K.P., Zhang Z., Zhang G.M., Zhang Y.L., Wang F. (2020). Analysis of DNA methylation profiles during sheep skeletal muscle development using whole-genome bisulfite sequencing. BMC Genom..

[15] Tan B.H., Hong L.J., Xiao L.Y., Wu J.J., Lu G.Y., Wang S.S., Liu L.Q., Zheng E.Q., Cai G.Y., Li Z.C. (2025). Rewiring of 3D chromatin topology orchestrates transcriptional reprogramming in muscle fiber-type specification and transformation. Nat. Commun..

[16] Yue C.J., Wang J.K., Shen Y.F., Zhang J.L., Liu J., Xiao A.P., Liu Y.S., Eer H., Zhang Q.E. (2023). Whole-genome DNA methylation profiling reveals epigenetic signatures in developing muscle in Tan and Hu sheep and their offspring. Front. Vet. Sci..

[17] Li Q., Hermanson P.J., Springer N.M. (2018). Detection of DNA Methylation by Whole-Genome Bisulfite Sequencing. Methods Mol. Biol..

[18] Derbala D., Garnier A., Bonnet E., Deleuze J.F., Tost J. (2024). Whole-Genome Bisulfite Sequencing Protocol for the Analysis of Genome-Wide DNA Methylation and Hydroxymethylation Patterns at Single-Nucleotide Resolution. Methods Mol. Biol..

[19] Greenberg M.V.C., Bourc’his D. (2019). The diverse roles of DNA methylation in mammalian development and disease. Nat. Rev. Mol. Cell Biol..

[20] Li D., Da L., Tang H., Li T., Zhao M. (2008). CpG methylation plays a vital role in determining tissue- and cell-specific expression of the human cell-death-inducing DFF45-like effector A gene through the regulation of Sp1/Sp3 binding. Nucleic Acids Res..

[21] Lee S.H., Kim J.M., Ryu Y.C., Ko K.S. (2016). Effects of Morphological Characteristics of Muscle Fibers on Porcine Growth Performance and Pork Quality. Korean J. Food Sci. Anim. Resour..

[22] Kim G.D., Ryu Y.C., Jeong J.Y., Yang H.S., Joo S.T. (2013). Relationship between pork quality and characteristics of muscle fibers classified by the distribution of myosin heavy chain isoforms. J. Anim. Sci..

[23] Van Laere A.S., Nguyen M., Braunschweig M., Nezer C., Collette C., Moreau L., Archibald A.L., Haley C.S., Buys N., Tally M. (2003). A regulatory mutation in IGF2 causes a major QTL effect on muscle growth in the pig. Nature.

[24] Bassel-Duby R., Olson E.N. (2006). Signaling pathways in skeletal muscle remodeling. Annu. Rev. Biochem..

[25] Murgia M., Serrano A.L., Calabria E., Pallafacchina G., Lomo T., Schiaffino S. (2000). Ras is involved in nerve-activity-dependent regulation of muscle genes. Nat. Cell Biol..

[26] Watt K.I., Goodman C.A., Hornberger T.A., Gregorevic P. (2018). The Hippo Signaling Pathway in the Regulation of Skeletal Muscle Mass and Function. Exerc. Sport Sci. Rev..

[27] Cisternas P., Henriquez J.P., Brandan E., Inestrosa N.C. (2014). Wnt Signaling in Skeletal Muscle Dynamics: Myogenesis, Neuromuscular Synapse and Fibrosis. Mol. Neurobiol..

[28] Glass D.J. (2005). Skeletal muscle hypertrophy and atrophy signaling pathways. Int. J. Biochem. Cell Biol..

[29] Yoon M.S. (2017). mTOR as a Key Regulator in Maintaining Skeletal Muscle Mass. Front. Physiol..

[30] Berdeaux R., Stewart R. (2012). cAMP signaling in skeletal muscle adaptation: Hypertrophy, metabolism, and regeneration. Am. J. Physiol.-Endocrinol. Metab..

[31] Dreiza C.M., Komalavilas P., Furnish E.J., Flynn C.R., Sheller M.R., Smoke C.C., Lopes L.B., Brophy C.M. (2010). The small heat shock protein, HSPB6, in muscle function and disease. Cell Stress. Chaperones.

[32] Sarparanta J., Jonson P.H., Vihola A., Luque H., Villar-Quiles R.N., Stojkovic T., Sian V., Walder C., Suominen T., Hackman P. (2026). C-terminal extension of HSPB6 in a family with myopathy and cataract. Hum. Mol. Genet..

[33] Wei H., Li Z.W., Wang X.B., Wang J., Pang W.J., Yang G.S., Shen Q.W. (2015). microRNA-151-3p Regulates Slow Muscle Gene Expression by Targeting ATP2a2 in Skeletal Muscle Cells. J. Cell. Physiol..

[34] Llansó L., Ravenscroft G., Aceituno C., Gutiérrez A., Parmar J., Gallano P., Caballero-Ávila M., Carbayo Á., Vesperinas A., Collet R. (2025). A Homozygous ATP2A2 Variant Alters Sarcoendoplasmic Reticulum Ca^2+^-ATPase 2 Function in Skeletal Muscle and Causes a Novel Vacuolar Myopathy. Neuropathol. Appl. Neurobiol..

[35] Malaichamy S., Idoux R., Polavarapu K., Sikic K., Holla E., Thompson R., Spendiff S., Schaenzer A., Kuesters B., Freeman E. (2025). Dominant rhabdomyolysis linked to a recurrent ATP2A2 variant reducing SERCA2 function in muscle. Brain.

[36] Sheng J.J., Jin J.P. (2016). TNNI1, TNNI2 and TNNI3: Evolution, regulation, and protein structure-function relationship. Gene.

[37] Lin X., Yan M., Yu W., Ma Y., Zhang L., Wei W., Chen J. (2021). Hoxa11 and Hoxa13 facilitate slow-twitch muscle formation in C2C12 cells and indirectly affect the lipid deposition of 3T3-L1 cells. Anim. Sci. J..

[38] Yamamoto M., Kuroiwa A. (2003). Hoxa-11 and Hoxa-13 are involved in repression of MyoD during limb muscle development. Dev. Growth Differ..

[39] Zhou Z.T., Lin Z.J., Wang M.R., Wang L.F., Ji Y.Q., Yang J., Yang Y.C., Zhu G.H., Liu T. (2024). Identification and verification of PTPN3 as a novel biomarker in predicting cancer prognosis, immunity, and immunotherapeutic efficacy. Eur. J. Med. Res..

[40] Varley K.E., Gertz J., Bowling K.M., Parker S.L., Reddy T.E., Pauli-Behn F., Cross M.K., Williams B.A., Stamatoyannopoulos J.A., Crawford G.E. (2013). Dynamic DNA methylation across diverse human cell lines and tissues. Genome Res..

[41] Krueger F., Andrews S.R. (2011). Bismark: A flexible aligner and methylation caller for Bisulfite-Seq applications. Bioinformatics.

[42] Langmead B., Salzberg S.L. (2012). Fast gapped-read alignment with Bowtie 2. Nat. Methods.

[43] Zhao L., Sun M.-a., Li Z., Bai X., Yu M., Wang M., Liang L., Shao X., Arnovitz S., Wang Q. (2014). The dynamics of DNA methylation fidelity during mouse embryonic stem cell self-renewal and differentiation. Genome Res..

[44] Lister R., Mukamel E.A., Nery J.R., Urich M., Puddifoot C.A., Johnson N.D., Lucero J., Huang Y., Dwork A.J., Schultz M.D. (2013). Global epigenomic reconfiguration during mammalian brain development. Science.

[45] Park Y., Wu H. (2016). Differential methylation analysis for BS-seq data under general experimental design. Bioinformatics.

[46] Krzywinski M., Schein J., Birol I., Connors J., Gascoyne R., Horsman D., Jones S.J., Marra M.A. (2009). Circos: An information aesthetic for comparative genomics. Genome Res..

[47] Robinson J.T., Thorvaldsdottir H., Turner D., Mesirov J.P. (2023). igv.js: An embeddable JavaScript implementation of the Integrative Genomics Viewer (IGV). Bioinformatics.

