# Supplementary material for: Genome-Wide DNA Methylation Sequencing Identifies Methylation-Associated Genes Related to Porcine Skeletal Muscle Fiber Diversity
Source: Int J Mol Sci. 2026 Oct 14;27(20):9093. doi: 10.3390/ijms27209093 (PMC13705056; doi:10.3390/ijms27209093)
Supplement: Supplementary file 1 [file ijms-27-09093-s001.zip › Table S1.Sequencing data quality control and mapping statistics.pdf]

Table S1 Sequencing data quality control and mapping statistics

Sample name	Bf28	Bf35	Bf36	Sol28	Sol35	Sol36
Raw Reads	437,162,9	433,224,5	471,715,1	365,065,0	372,796,5	342,515,7
Raw Bases (G)	131.15	129.97	141.51	109.52	111.84	102.75
Clean Reads	428,706,0	428,658,3	465,398,5	359,285,1	366,643,7	336,219,4
Clean Bases (G)	120.46	123.31	133.35	102.73	105.02	95.67
Clean_ratio (%)	91.85	94.88	94.23	93.80	93.90	93.11
Q20 (%)	97.63	98.10	98.00	97.96	98.05	97.69
Q30 (%)	92.41	93.88	93.71	93.75	93.80	93.03
GC Content (%)	20.09	20.41	20.43	20.37	20.26	20.21
BS Conversion	98.91	98.98	98.42	99.13	99.53	99.48
Mapped reads	362,856,8	368,388,9	398,008,8	305,967,1	315,166,9	287,198,6
Mapping rate (%)	84.64	85.94	85.52	85.16	85.96	85.42
Duplication rate	37.73	33.05	37.30	32.91	30.91	36.54
sites_num	2,423,763	2,424,913	2,425,615	2,423,671	2,423,930	2,421,950
sites_covgMean	25.59	28.49	28.74	23.57	25.04	20.82
sites_numCovg1	96.88	96.92	96.95	96.87	96.88	96.80
sites_numCovg5	94.36	95.20	95.13	94.32	94.60	93.03
sites_numCovg1	85.61	89.38	88.94	85.28	86.68	80.69
C_covgMean	10.80	12.00	12.10	10.00	10.60	8.80
C (Mb)	11159.80	12441.00	12503.50	10394.60	10968.60	9145.30
CG (Mb)	494.30	560.60	561.90	471.40	493.80	407.20
CHG (Mb)	2280.10	2566.20	2569.50	2142.60	2259.90	1878.60
CHH (Mb)	8385.40	9314.10	9372.20	7780.50	8214.90	6859.60
meanC (%)	4.38	4.35	4.88	4.21	3.75	3.74
MeanCG (%)	70.57	70.72	70.70	69.58	69.38	68.82
MeanCHG (%)	1.36	1.26	1.84	1.13	0.68	0.72
MeanCHH (%)	1.30	1.20	1.77	1.10	0.65	0.70

Note: sites_num: the number of sites detected on the genome; sites_covgMean: average coverage depth of all sites in the genome; sites_numCovg1: the ratio of the number of bases greater than or equal to 1 times the depth of the genome sequenced to the total length of the genome; sites_numCovg5: the ratio of the number of bases greater than or equal to 5 times the sequencing depth of the genome to the total length of the genome; sites_numCovg10: the ratio of the number of bases greater than or equal to 10 times the depth of the genome sequenced to the total length of the genome; C_covgMean: The average coverage depth of all C sites in the whole genome; C (Mb): The number of bases mapped to the C sites of the whole genome; CG (Mb): the number of bases aligned to the cytosine in the CG region of the whole genome; CHG (Mb): the number of bases aligned to the cytosine in the CHG region of the whole genome; CHH (Mb): the number of bases aligned to the cytosine in the CHH region of the whole genome;

MeanC (%): The average methylation level of all C sites in the whole genome; MeanCG (%): The average methylation level of all C sites in the CG region; MeanCHG (%): The average methylation level of all C sites CHG region; MeanCHH (%): The average methylation level of all C sites CHH region.
