# Supplementary material for: Genome-Wide DNA Methylation Sequencing Identifies Methylation-Associated Genes Related to Porcine Skeletal Muscle Fiber Diversity
Source: Int J Mol Sci. 2026 Oct 14;27(20):9093. doi: 10.3390/ijms27209093 (PMC13705056; doi:10.3390/ijms27209093)
Supplement: Supplementary file 1 [file ijms-27-09093-s001.zip › Table S2.Summary of genome-wide methylation for each skeletal muscle.pdf]

Table S2 Summary of genome-wide methylation for each skeletal muscle

Samples	Bf28	Bf35	Bf36	Sol28	Sol35	Sol36
Total number of cytosines (Cs)	1,035,107,725	1,035,107,725	1,035,107,725	1,035,107,725	1,035,107,725	1,035,107,725
Number of methylated Cs (mCs)	37,667,145	41,661,421	40,711,047	37,366,389	38,487,554	32,788,008
mCs percent (%)	3.63%	4.02%	3.93%	3.60%	3.71%	3.16%
C in CG	61,239,920	61,239,920	61,239,920	61,239,920	61,239,920	61,239,920
mCs in CG	36,327,819	40,297,605	39,568,640	35,977,747	36,934,632	31,570,290
mCpG percent (%)	59.32%	65.80%	64.61%	58.74%	60.31%	51.55%
C in CHG	224,048,188	224,048,188	224,048,188	224,048,188	224,048,188	224,048,188
mC in CHG	306,420	326,792	278,569	292,177	326,279	275,113
mCHG percent (%)	0.13%	0.14%	0.12%	0.13%	0.14%	0.12%
C in CHH	749,819,617	749,819,617	749,819,617	749,819,617	749,819,617	749,819,617
mC in CHH	1,032,906	1,037,024	863,838	1,096,465	1,226,643	942,605
mCHH percent (%)	0.13%	0.13%	0.11%	0.14%	0.16%	0.12%
mC (%)	100%	100%	100%	100%	100%	100%
mCG (%)	96.44%	96.72%	97.19%	96.28%	95.96%	96.28%
mCHG (%)	0.81%	0.78%	0.68%	0.78%	0.84%	0.83%
mCHH (%)	2.74%	2.48%	2.12%	2.93%	3.18%	2.87%

Note: CG, CHH, and CHG represent different context, where H is A, C, or T.
